# Interactions between Tick-Borne Encephalitis Virus Nonstructural Protein 1 and Blood–Brain Barrier Tight Junction Proteins: Potential Clues to Strain-Specific Neuropathogenicity

**DOI:** 10.1021/acsomega.6c00564

**Published:** 2026-09-22

**Authors:** Jarinya Chaopreecha, Jordan Liburd, Shahid Khalid, Songeziwe Ntsimango, Ian M. Jones, Jennifer H. Tomlinson, Niluka Goonawardane

**Affiliations:** † School of Molecular and Cellular Biology Faculty of Biological Sciences, 4468 University of Leeds, Leeds LS2 9JT, U.K.; ‡ Molecular Sciences Institute, School of Chemistry, 37707 University of the Witwatersrand, PO Wits, Johannesburg 2050, South Africa; § School of Biological Sciences, University of Reading, Reading RG6 6UR, U.K.; ∥ Astbury Centre for Structural Molecular Biology, School of Molecular and Cellular Biology Faculty of Biological Sciences, 4468 University of Leeds, Leeds LS2 9JT, U.K.; ⊥ Department of Biology, 6396 University of Oxford, Life and Mind Building, South Parks Road, Oxford OX1 3EL, U.K.

## Abstract

Tick-borne encephalitis virus (TBEV) invades the central nervous system (CNS) through strain-specific mechanisms that remain poorly understood. For mosquito-borne orthoflaviviruses such as dengue and yellow fever viruses, the nonstructural protein 1 (NS1) has been shown to disrupt endothelial barrier integrity by targeting tight junction proteins (TJPs), facilitating viral neuroinvasion. However, comparable mechanisms in TBEVs remain largely unexplored. Here, we investigated the potential interaction of NS1 from high (Hypr)- and low (Vs)-pathogenic TBEV strains with different blood–brain barrier (BBB) TJPs, using AlphaFold3 (AF3) multimer modeling and *in vitro* binding assays. AF3 modeling suggested that NS1 from the highly pathogenic strain may interact with multiple TJPs, including junctional adhesion molecule A (JAM-A), a key component of the paracellular barrier. In contrast, the low-pathogenic Vs showed a more restricted interaction profile. Experimental validation using recombinant NS1 proteins revealed strain-specific binding profiles: Hypr NS1 displayed high-affinity, saturable direct binding to immobilized JAM-A (K_D,app_ = 0.81 nM), whereas Vs NS1 showed no binding. Immunofluorescence assays on human lung epithelial (A549) and BBB (hCMEC/D3) cells further demonstrated colocalization between Hypr NS1 and JAM-A, supporting a potential interaction. No direct binding to ZO-1, a barrier scaffold lacking an extracellular domain, was observed for either strain. This differential interaction profile may be modulated by 22 amino acids localized to the Wing and β-Ladder domains within NS1, which distinguish Hypr from Vs. Notably, despite its limited interactions with the investigated TJPs, the Vs strain is associated with slow-progressive infections that can culminate in chronic neurological disease. This highlights the need for further studies into noncanonical pathways of neuroinvasion.

## Graphical abstract

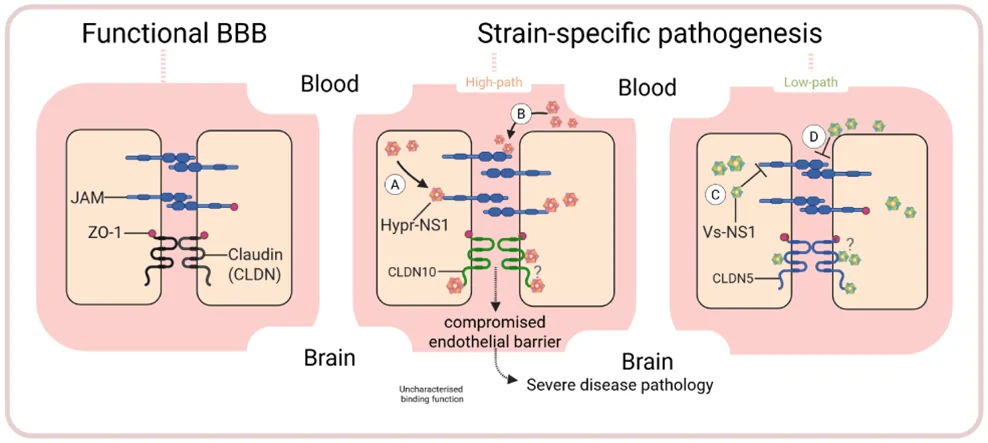

## Introduction

Orthoflaviviruses, an important genus within the *Flaviviridae* family, include medically significant mosquito- and tick-borne viruses such as dengue (DENV), West Nile (WNV), Zika (ZIKV), and tick-borne encephalitis viruses.^1^ All share structural and pathogenic features and cause a wide spectrum of clinical outcomes in humans ranging from asymptomatic infections to severe, sometimes fatal disease. Severe flavivirus infections are associated with systemic vascular leakage, hemorrhagic manifestations, or encephalitic pathology,^2–5^ often involving breach or compromise of the blood–brain barrier (BBB).^6–8^

The orthoflavivirus genome encodes three structural proteins (C, prM/M, and E) and seven nonstructural (NS) proteins (NS1, NS2A, NS2B, NS3, NS4A, NS4B, and NS5). NS1 is a structurally conserved and critical virulence factor encoded by orthoflaviviruses, including TBEV. It is a glycoprotein that exists in multiple forms, intracellular, membrane-associated, and secreted, which circulates in the host bloodstream^9^ (Figure 1A)—all of which contribute to virus replication, immune evasion, and pathogenesis.^10–12^

**Figure 1:**
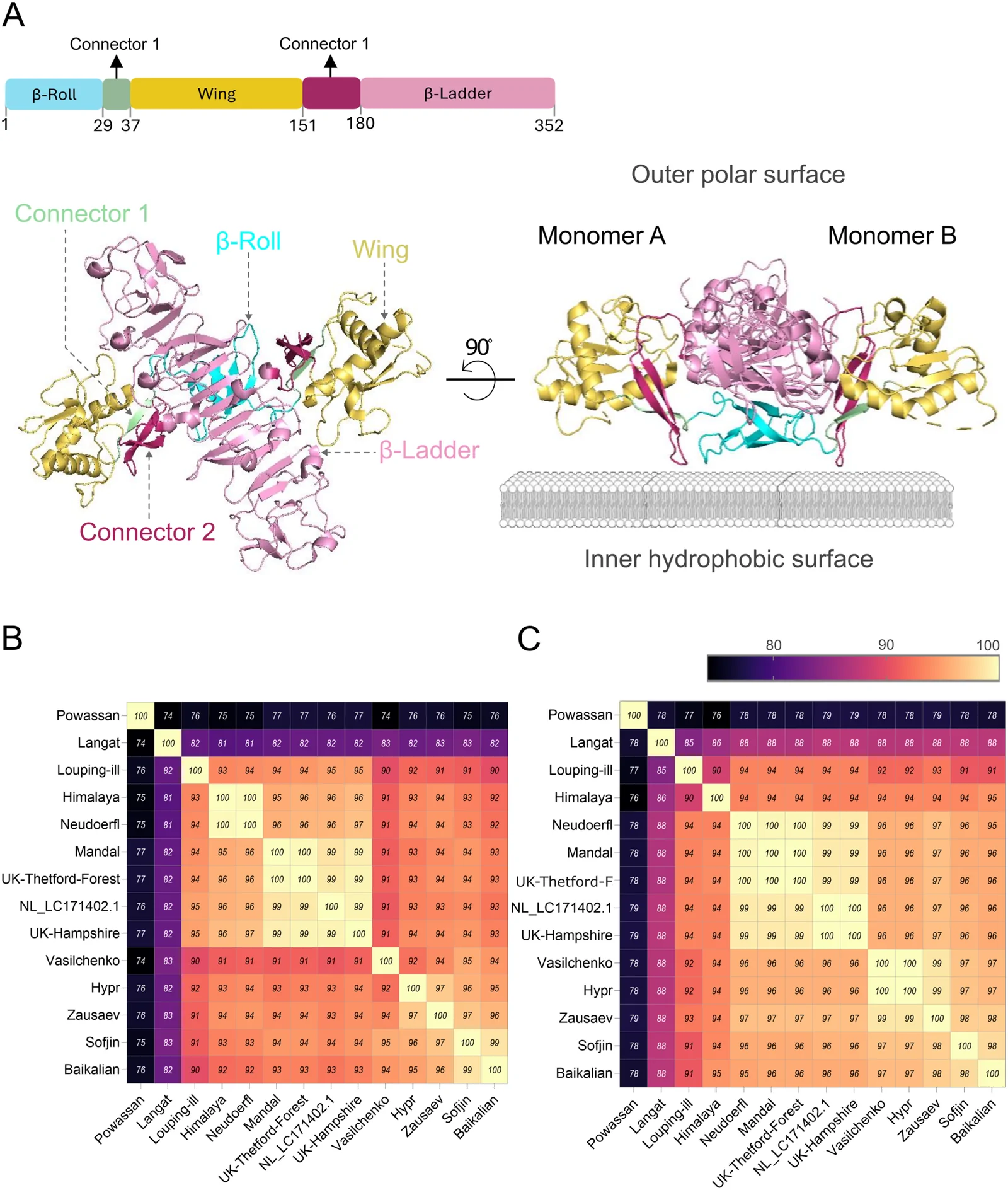
Cryo-EM structure of orthoflavivirus non-structural protein 1 (NS1). (A) The NS1 dimer is shown in cartoon representation. The ribbon diagram highlights the β-roll, wing, β-ladder, and connector domains (1 and 2), colored blue, yellow, pink, green, and purple, respectively, in a single monomer. Dengue virus (DENV) NS1 dimer (PDB ID: 4OIG) is shown interacting with a phospholipid membrane. Percentage identity matrices for NS1 (B) or (C) (envelop) E proteins are indicated among 14 tick-borne orthoflaviviruses (TBFV), generated by ClustalW. Each matrix represents the percentage of identical residues between each pair of sequences in the alignment. Identity values are shown as an inferno heatmap with a gradient from highest (light yellow; 100%) to lowest (dark purple; 74%).

Extensive work in mosquito-borne orthoflaviviruses, particularly DENV, yellow fever virus (YFV), and ZIKV, demonstrates that secreted NS1 directly perturbs endothelial barrier integrity by engaging glycocalyx-associated molecules, inducing hyperpermeability, and disrupting tight junction protein (TJP) architecture through complement activation, inflammatory signaling, and direct endothelial binding.^13–15^ These studies collectively show that NS1 functions as a soluble pathogenic effector capable of driving vascular dysfunction independently of viral replication and highlight its capacity to remodel endothelial surfaces, destabilize intercellular junctions, and facilitate barrier breakdown.^13,15^

In contrast to the depth of mechanistic insight available for mosquito-borne orthoflaviruses, the role of NS1 in tick-borne arbovirus biology remains comparatively underexplored. This gap is particularly important given that TBEV is a neurotropic arbovirus that can cause severe neurological disorders,^16–18^ including encephalitis and meningitis,^19–21^ often leading to long-term neurological sequelae.^22,23^ A defining feature of TBEV pathogenesis is its capacity to breach the BBB and trigger neuroinflammation in a strain-dependent manner.^24–26^ Recent evidence implicates NS1 as a key virulence factor in endothelial dysfunction and immune modulation.^14,15,27,28^ In related viruses (including DENV and YFV), secreted NS1 (sNS1) has been shown to compromise vascular integrity by targeting tight junction components such as ZO-1 and claudins, promoting vascular leakage and amplifying inflammatory responses.^13^ Despite these parallels, the contribution of TBEV NS1 to BBB disruption remains poorly defined, and direct mechanistic insights are lacking. Addressing these knowledge gaps is critical for unraveling the pathogenesis of TBEV and developing targeted therapeutics.

In this study, we investigated the potential interactions between NS1 proteins from high- and low-pathogenicity TBEV strains and TJPs to explain the observed functional consequences at the BBB. Using the AlphaFold3 (AF3) multimer model,^29,30^ an advanced deep learning framework that significantly enhances the accuracy of multimeric protein complex predictions, we identified strain-specific binding patterns between TBEV NS1 and the TJP JAM-A and validated these interactions *in vitro*. These findings offer insights into how NS1 may contribute to endothelial barrier disruption during TBEV infection and highlight the importance of this axis. Further *in vivo* validation is required to better understand the underlying mechanisms.

## Methods

### Recombinant TBEV NS Protein Generation and Purification

N-terminally His-tagged Vs (accession number: AF069066.1) or Hypr (accession number: U39292.1) NS1 was heterologously expressed in *E. coli* BL21 Gold cells harboring the pET-28a-TEV-NS1 plasmid (synthesized by GenScript). Protein was expressed in LB medium containing 50 μg/mL kanamycin. Briefly, cultures were incubated at 37 °C, with shaking at 200 rpm until an OD_600 nm_ of ∼0.6 was reached. Protein expression was induced with 1 mM IPTG and cultures were incubated at 25 °C overnight with shaking at 200 rpm. Cells were harvested by centrifugation at 4500 × *g* for 30 min at 4 °C. Cell pellets were resuspended in 50 mM Tris-HCl, 500 mM NaCl, 2 mM MgCl_2_, 10 mM imidazole, 10% v/v glycerol, pH 8.0, containing Benzonase (Merck) and cOmplete EDTA-free protease inhibitors (Roche), and lysed by sonication on ice prior to centrifugation at 11,000 × *g* at 4 °C for 20 min. NS1 was located in the insoluble fraction and recovered via on-column refolding. The insoluble fraction was resuspended in denaturing buffer (20 mM Tris-HCl, 300 mM NaCl, 20 mM imidazole, 5 M guanidine HCl, 10 mM DTT, pH 8.0) and incubated on ice for 1 h before centrifugation at 13,000 × *g* for 20 min at 4 °C. Extracted proteins were added to a Ni-NTA column (Qiagen) and washed with denaturing buffer. A gradient wash was performed, gradually reducing the guanidine HCl and DTT concentrations to 0 M. The column was further washed in refolding buffer (20 mM Tris-HCl, 300 mM NaCl, 20 mM imidazole, pH 8.0). Protein was eluted in buffer (20 mM Tris-HCl, 300 mM NaCl, 500 mM imidazole, pH 8.0) and purity was assessed using SDS-PAGE. Purified NS1 was exchanged into 50 mM Tris-HCl, 500 mM NaCl, 2 mM MgCl_2_, 10 mM imidazole, 10% v/v glycerol, pH 8.0, and stored at −80 °C.

### Circular Dichroism (CD)

Successful refolding was assessed using CD spectroscopy as described previously^31,32^ and compared to commercially available NS1. Briefly, CD measurements were performed using a JASCO (model J-715) spectropolarimeter. Spectra were recorded in 0.1 cm path-length quartz cells at a protein concentration of 2 μM in 10 mM phosphate buffer at pH 8.0. Three consecutive scans were compiled (180–260 nM) and average spectra were stored. Observed raw ellipticities were converted to molar ellipticity (θ). Secondary structures were estimated from fitted far-UV spectra using the DichroWeb server (http://dichroweb.cryst.bbk.ac.uk/html/home.shtml).

### Multiple Sequence Alignment and Percent Identity Matrix Generation

NS1 protein sequences of TBEV isolates and closely related tick-borne orthoflaviviruses were downloaded from the NCBI (Vasilchenko [accession number; AF069066.1], Hypr [U39292.1], Neudoerfl [U27495.1], Zausaev [AF527415.1], UK-Thetford-Forest [MN128700.1], UK-Hampshire-2019 [MN661145.1], Sofjin-Ru [JN229223.1], Mandal-2009 [KF991107.1], NL [LC171402], Baikalian [EF469661.1], Himalaya-1 [MG599476.1], and other closely related TBFV (Louping-ill-virus [Y07863.1], Langat-virus_[NC_003690.1] and Powassan virus [NC_003687.1]) and aligned using ClustalW, as implemented in BioEdit (Figure 2).

**Figure 2:**
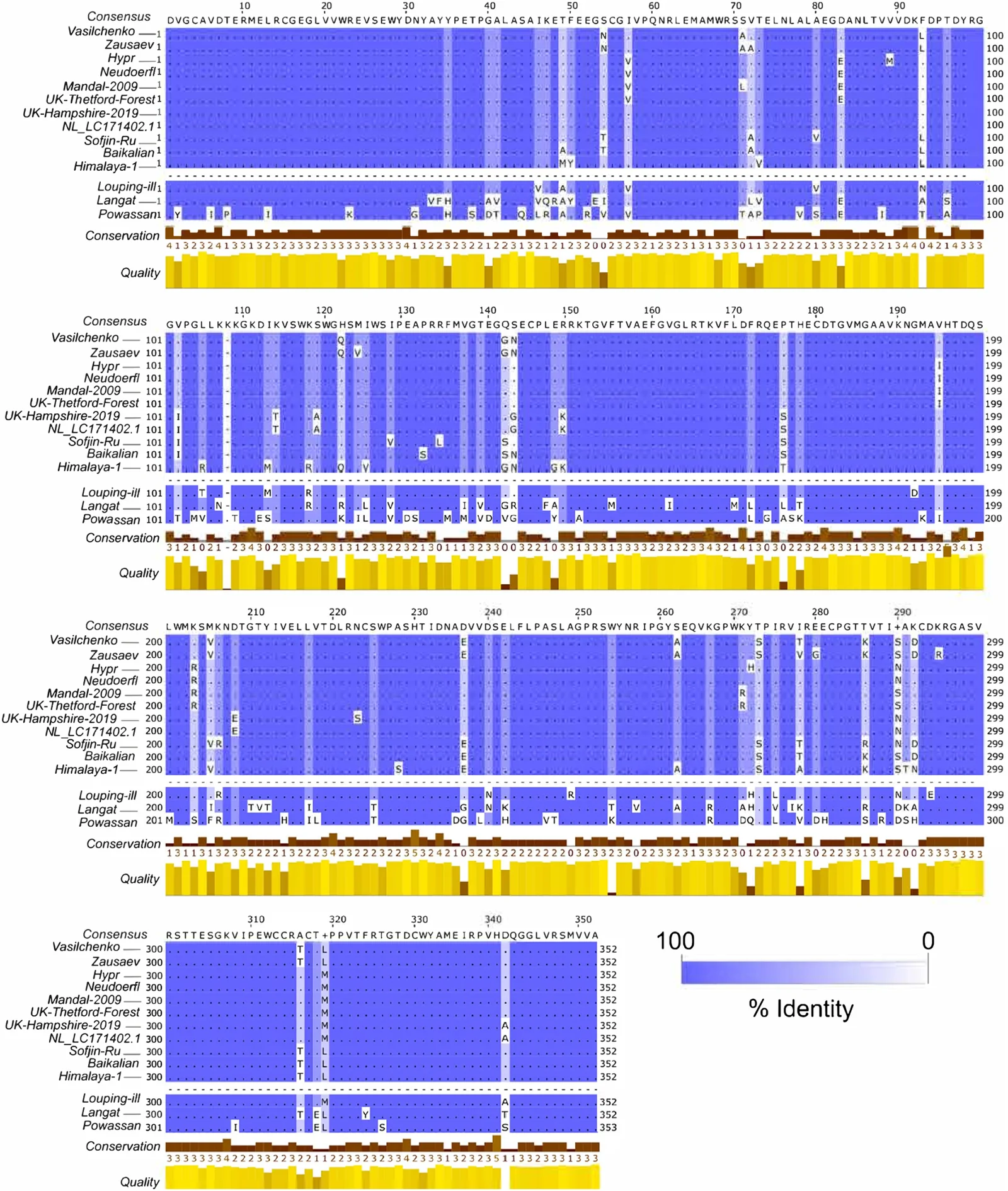
Sequence alignment of NS1 proteins from tick-borne orthoflaviviruses (TBFV). NS1 proteins of different TBFVs were compared using a multiple alignment tool, CLUSTAL Omega. This showed conserved regions among the NS1 sequences, indicated as percentage (%) sequence identity from highlighted areas of similarity (100%; dark purple) to dissimilarity (0; white). The consensus for all 14 sequences is indicated at the top. Alignments are grouped into TBEV isolates (Vasilchenko [accession number; AF069066.1], Hypr [U39292.1], Neudoerfl [U27495.1], Zausaev [AF527415.1], UK-Thetford-Forest [MN128700.1], UK-Hampshire-2019 [MN661145.1], Sofjin-Ru [JN229223.1], Mandal-2009 [KF991107.1], NL [LC171402], Baikalian [EF469661.1], Himalaya-1­[MG599476.1], and other closely related TBFVs (Louping-ill-virus [Y07863.1], Langat-virus_[NC_003690.1], and Powassan [NC_003687.1]. Sequence conservation of total alignments with less than 20% of gaps and BLOSUM62 alignment quality are shown.

Default parameters were used unless otherwise specified, including the Gonnet substitution matrix, a gap-opening penalty of 10, and a gap-extension penalty of 0.2. Following alignment, a percent identity matrix (PIM) was generated, which calculates the pairwise percentage of identical residues between aligned sequences. This matrix provided a quantitative measure of sequence similarity and was used to assess evolutionary relationships and sequence conservation.

### NS1–BBB TJP Interaction Predictions and Interface Analysis by AlphaFold3 (AF3)

Predicted interactions between TBEV Hypr or Vs NS1 and host TJPs were modeled using AF3.^29,30^ AF3 structural predictions were visualized using PyMOL^33^ (https://pymol.org/2/). Briefly, the NS1–TJP complex was loaded into PyMOL, and residues at the protein–protein interface were selected manually based on proximity and surface contact. The “find polar contacts” function was used to detect potential hydrogen bonds and salt bridges between the proteins. Interacting residue pairs were further refined by selecting only those forming direct contacts between NS1 and TJP.

Distances between interacting atoms were measured using the “distance” measurement tool in PyMOL. All interacting residue pairs were ranked based on the shortest interatomic distance. Top-ranking interactions (those with the shortest and most structurally significant contacts) were visualized and annotated in the PyMOL structural model. A complete list of all detected interactions, including residue identities and distances, is shown in Supplementary Tables S3–S5.

### Molecular Docking and Thermodynamic Analysis

NS1–TJP docking was performed using the HDOCK web server (http://hdock.phys.hust.edu.cn/), which uses a hybrid scoring function combining template-based modeling and free docking algorithms. Homodimers of NS1 proteins from TBEV Hypr and Vs strains were docked against several human full-length proteins or extracellular domains, including Claudin-5, Claudin-10, JAM-A, JAM-B, and JAM-C. Docking scores were recorded as a proxy for binding energy, with higher negative scores indicating stronger predicted interactions. Docking was performed in the default mode, allowing the server to automatically identify potential binding sites and generate a ranked list of predicted complex models.

To estimate binding affinities under physiological (25 °C, 298.15 K) and febrile (40 °C, 313.15 K) conditions, Gibbs free energy (ΔG) and equilibrium dissociation constants (K_D_) were calculated from docking scores using the thermodynamic equation:

$$\Delta G=-RT\;\ln{\;K}_{D}\Rightarrow K_{D}=e^{-\Delta G/RT}$$

where R is the gas constant (0.001987 kcal·mol^–1^·K^–1^) and T is the temperature in Kelvin. We assumed docking scores approximate ΔG in kcal/mol, consistent with prior benchmarks for HDOCK scoring.^34,35^ Fold changes in K_D_ between 25 °C and 40 °C were computed to assess temperature sensitivity (Supplementary Table S1).

### Enzyme-Linked Immunosorbent Assay (ELISA) for NS1–JAM-A/ZO-1 Interaction

High-binding 96-well plates were coated with recombinant human JAM-A (Kactus, HM10A) or ZO-1 (Invitrogen, RP-99371) at a concentration of 1 μg/mL in 0.1 M sodium bicarbonate buffer (pH 9.6) and incubated for 2 h at room temperature (RT). Wells were washed three times with PBS containing 0.1% Tween-20 (PBS-T), followed by blocking with 5% bovine serum albumin (BSA) in PBS supplemented with 0.1 mM EDTA for 1 h at RT. Recombinant NS1 proteins from TBEV Hypr or Vs strains (variable starting concentrations as indicated: 0.5, 1, or 2 μg/mL) were 2-fold serially diluted and incubated for 2 h at RT. For comparison, Hypr NS1 produced in mammalian HEK293 cells (Native Antigen; starting concentrations 0.25, 0.5, 1, or 2 μg/mL) was analyzed under the same experimental format (96-well) and conditions to assess NS1–JAM-A binding. After washing (3× with PBS-T), bound NS1 was detected using mouse anti-TBEV NS1 monoclonal antibodies (R&D Systems, MAB10106; 0.05 μg/mL), followed by incubation with HRP-conjugated anti-mouse IgG (Sigma, A9044; 1:40,000) for 1 h at RT. Plates were washed and incubated with TMB peroxidase substrate (Sigma, T5569) in the dark for up to 30 min at RT. The reaction was stopped with 1 M hydrochloric acid. Absorbance was measured at 450 nm using a NanoQuant Infinite M200 plate reader (Tecan).

Raw OD_450_ values from technical replicates were averaged and analyzed using Python (SciPy, NumPy, and Matplotlib). NS1 concentrations were converted from mass concentrations (μg mL^–1^) to molar concentrations (M) with a molecular mass of 48 kDa for recombinant NS1. Apparent equilibrium dissociation constants (K_D,app_) were estimated by nonlinear least-squares regression on the untransformed molar concentrations using a one-site specific binding model:

$$Y=\left(B_{\max}\times \left[\mathrm{NS}1\right]\right)/\left(K_{D,\mathrm{app}}+\left[\mathrm{NS1}\right]\right)$$

where Y is the measured OD_450_, B_max_ is the maximum binding response, [NS1] is the untransformed molar concentration, and K_D,app_ is the apparent equilibrium dissociation constant. Nonlinear regression was performed using the SciPy curve_fit function. Concentration–response curves were displayed on a log_10_ (molar concentration) axis for graphical presentation only; logarithmic transformations were not used for parameter estimation. For ZO-1 experiments, concentration–response data showed no saturable binding suitable for equilibrium binding analysis. Consequently, K_D,app_ values were not determined. Data are presented as mean ± SD with descriptive trend lines.

For statistical analysis, OD_450_ values obtained at selected NS1 concentrations (0.25 μg/mL and 1 μg/mL) were compared between Hypr and Vs NS1 using an unpaired two-tailed Student’s *t*-test. Statistical significance was defined as *p* < 0.05.

Plots were generated using Matplotlib in Python. Concentration–response curves are presented as the mean ± SD with the corresponding nonlinear regression fit (JAM-A) or descriptive trend lines (ZO-1). Bar graphs show the mean ± SD for the indicated NS1 concentrations.

### Immunofluorescence (IF) Analysis of Replicon-Transfected Cells

Hypr and Vs replicon systems have been described elsewhere.^36^ Briefly, structural genes (nts 183-2386) were removed to make them noninfectious. The 5′UTR sequence and Stem-loops (SLs) 3 and 4 were retained in the C-terminus of the C-protein due to their requirement during the formation of secondary RNA structures that are essential for genome cyclization. Luciferase was cloned downstream of the 5′-end of the C-protein to provide a marker of both replication and translation. To ensure correct polyprotein processing and NS1 translocation to the ER, a ribosome-stuttering 2A peptide from foot-and-mouth disease virus (FMDV) was incorporated downstream of the luc-cu sequence prior to the transmembrane (TM) region of E (22 codons).^37^

For IF analysis, replicon-transfected hCMEC/D3 (SCC066; Merck) cells were cultured on rat-tail collagen (Gibco, USA)-coated 8-well chambered coverglass slides (Merck), and A549 cells were cultured on uncoated 8-well chambered coverglass slides (Corning, USA). Cells were fixed with 4% paraformaldehyde (Applichem GmbH) and permeabilized in PBS-T (0.1% vol/vol Triton X-100 in PBS) for 5 min. Cells were blocked in PBS-T containing 5% wt/vol bovine serum albumin (BSA) for 10 min and probed with primary anti-TBEV NS1 (R&D Systems, MAB10106; 1:100) and anti-human JAM-A (CD321; Invitrogen) at 1:100 in PBS-T containing 1% wt/vol BSA for 1 h at RT. Cells were washed in PBS and stained with 1:500 fluorochrome-conjugated secondary antibodies (Thermo Fisher) in PBS-T containing 1% wt/vol BSA for 1 h at RT in the dark. Confocal microscopy images were acquired on a Zeiss LSM880 microscope with a Airyscan or an Olympus FV3000. Postacquisition analysis was performed using Zen software (Zen version 2015 black edition 2.3, Zeiss) or Fiji (v1.49) software.^38^

### Image Analysis

Images were analyzed using the Fiji package for ImageJ. For colocalization analyses, Manders’ overlap coefficients were calculated using Fiji software with the Just Another Co-localization Plugin (JACoP) (National Institutes of Health),^39,40^ where the M1 coefficient reports the fraction of the NS1 signal that overlaps with the JAM-A signal, whilst the M2 coefficient reports the fraction of the JAM-A signal overlapping with the NS1 signal. Coefficient values ranged from 0 to 1, corresponding to nonoverlapping images and 100% colocalization, respectively. Colocalization was assessed on ≥120 cells from a minimum of two independent experiments.

### Transepithelial/Transendothelial Electrical Resistance (TEER) Measurements

TEER was used to evaluate the integrity and barrier properties of A549 and hCMEC/D3 monolayers cultured on Transwell inserts (Corning). Prior to seeding, inserts used for hCMEC/D3 cultures were coated with rat-tail collagen (Gibco) to promote cell attachment. At 24 h postseeding, the culture medium was replaced with prewarmed Hanks’ Balanced Salt Solution (HBSS) in both apical and basolateral compartments. Inserts were equilibrated for 10 min at 37 °C in a humidified atmosphere containing 5% CO_2_ prior to assessment. TEER measurements were performed using an EVOM2 epithelial voltohmmeter (World Precision Instruments, Sarasota, FL, USA) equipped with STX electrodes, according to the manufacturer’s instructions. Briefly, the shorter electrode was placed in the apical compartment and the longer electrode in the basolateral compartment of each insert (MilliporeSigma, USA) and electrical resistance was recorded. Blank inserts containing HBSS alone were measured in parallel, and values were subtracted from cell-seeded inserts to account for the intrinsic resistance of the membrane and medium. TEER values were normalized to the surface area of the Transwell membrane and expressed as Ω·cm^2^. Measurements were obtained 24 h post-seeding for both cell types.

### Statistical Analysis

Statistical significance was determined in GraphPad Prism using a Student’s *t*-test with Welch’s correction or a one-way ANOVA with Bonferroni’s correction unless otherwise stated. Where applicable, a two-way ANOVA was used to assess interactions between strain and concentration, with a Bonferroni correction applied for multiple testing. Thresholds for statistical significance were set at: *p* < 0.05 (*), *p* < 0.01 (**), *p* < 0.001 (***), *p* < 0.0001 (****). All data are presented as the mean ± SEM unless otherwise stated. The number of biological replicates used in each experiment is specified in the corresponding figure legends.

## Results

### Relative Diversity of NS1 Protein among Tick-Borne Orthoflaviviruses

To investigate the evolutionary relationships among tick-borne orthoflaviviruses (TBFVs), we generated pairwise percentage-identity matrices based on NS1 protein sequences from 14 viral isolates (Figure 1B). This panel included multiple TBEV strains, including recent isolates from the UK (Figure 2) (Thetford-Forest [MN128700.1], Hampshire-2019 [MN661145.1]), and the Netherlands (NL [LC171402]), as well as more distantly related viruses, including Powassan virus (POWV; NC_003687.1), Langat virus (LGTV; NC_003690.1), and Louping-ill virus (LIV; Y07863.1). The resulting identity values were visualized as a heatmap (Figure 1B), revealing high conservation within the broader TBEV group, with pairwise identities generally exceeding 92%. In contrast, interspecies comparisons showed substantially lower identity values, with Powassan virus sharing only ∼74–77% identity and Langat virus sharing 81–83% identity with TBEV strains, consistent with their divergent evolutionary trajectories.

In contrast, the envelope (E) protein, which mediates host cell entry and serves as a major antigenic determinant, exhibited less pronounced subtype-specific divergence (Figure 1C). Within the TBEV group, E protein identities ranged from 94% to 100%. Notably, Hypr and Vasilchenko shared 100% identity, indicating limited sequence divergence. Sofjin, Zausaev, and Baikalian exhibited similarly high levels of conservation. Comparisons with more distantly related orthoflaviviruses, such as Powassan virus and Langat virus, revealed lower E protein identities (77–88%), in line with their antigenic profiles. Collectively, these data suggest that whilst both NS1 and E proteins are conserved across TBEV, NS1 may be subject to distinct selective pressures, potentially reflecting differences in functional constraints linked to pathogenicity.

### Structural Comparison of NS1 across Orthoflavivirus Strains Reveals Lineage- and Pathogenicity-Specific Differences

To investigate the structural basis underlying the differences in pathogenicity and vector specificity among orthoflaviviruses, we performed a comparative structural analysis of NS1 from representative tick-borne (TBEV) and mosquito-borne (ZIKV, JEV, and WNV) viruses. We focused on identifying lineage-specific amino acid (AA) differences and structural features that may correlate with pathogenic potential and tick- or mosquito-vector tropism.

Similar to other orthoflaviviruses, the NS1 protein of TBEV comprises three distinct domains: a β-roll (residues 1–29), a wing domain (residues 38–180), and a β-ladder domain (residues 181–352) (Figure 1A).^10,11^ High-resolution structural alignment of NS1 monomers from the TBEV-Vs (low-pathogenic) and -Hypr (high-pathogenic) strains revealed several discrete (22 AA) substitutions, particularly localized in the surface-exposed wing domain (9 AA) and the distal β-ladder (13 AA) domain (Figure 3A, Supplementary Table S1). This domain clustering, with no AA changes in either the β-roll or connector regions, may contribute to the differences in neurovirulence observed between these strains.

**Figure 3:**
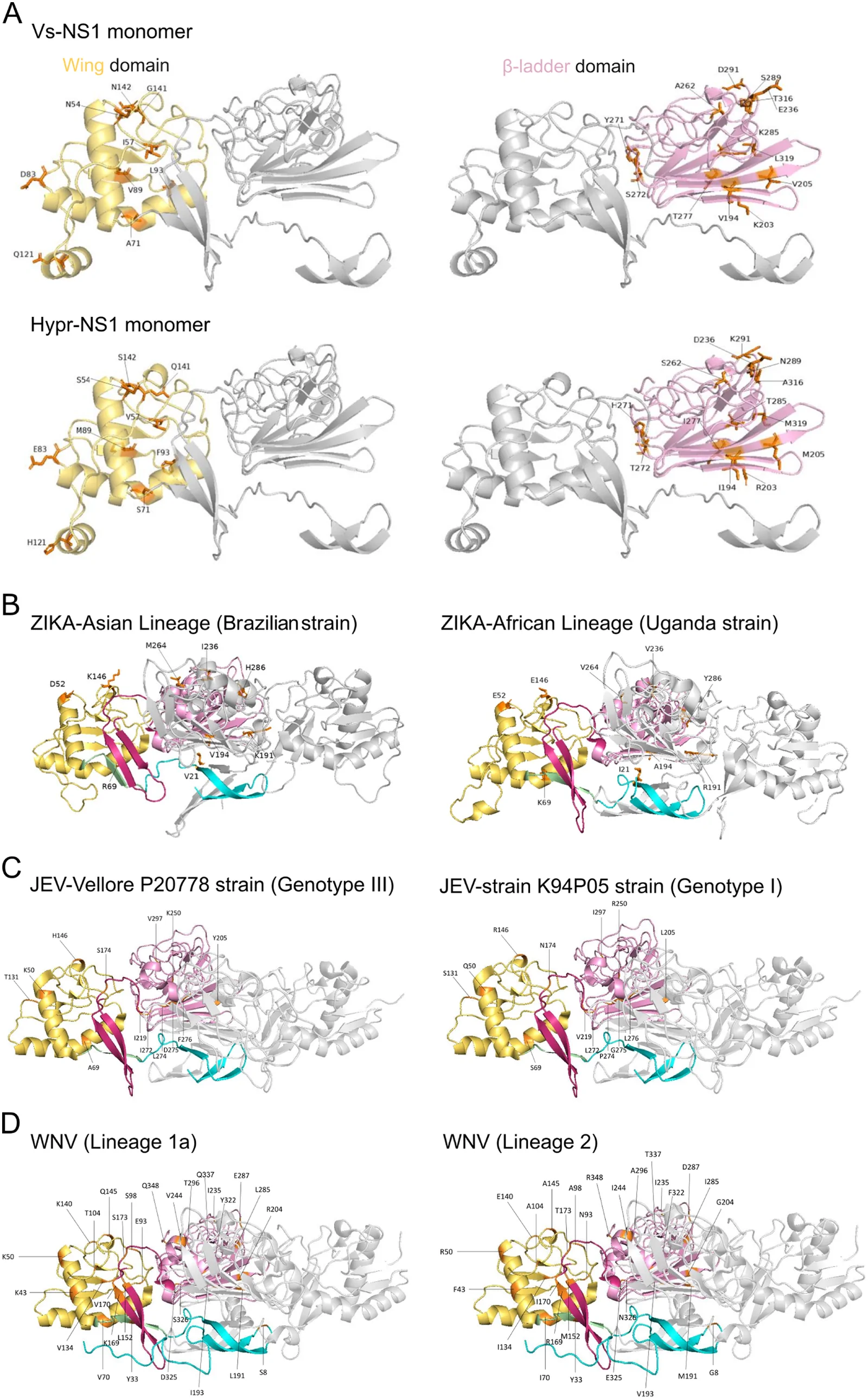
Localization of sequence changes in representative low- and high-pathogenic strains of TBEV (A), ZIKA (B), JEV (C), and WNV (D) NS1. Domains are color coded as in Figure 1A. Initial atomic positions were based on (PDB ID: 4OIG) for all. (A) Positions of 22-AA changes in the three-dimensional structure of the Vs (low-path)- and Hypr (high-path)- NS1 monomer shown by AlphaFold3 and visualized in PyMOL. A focused view of the mutations in the Wing (yellow) and β-ladder (Pink) domains is indicated. Mutated amino acids are shown as an atomic stick representation. (B–D) NS1 of high- and low-pathogenic strains are indicated left and right, respectively. Domains are color coded as in Figure 1A. (B) Dimer of Asian (PDB: 5GS6) (left) and African (PDB: 5K6K) ZIKA (right) virus NS1, showing all 9 AA changes (orange). (C) Dimer of JEV genotype III- and genotype I-NS1 with 13 site-specific amino acid changes highlighted in orange. (D) NS1 dimer of lineage 1a- and lineage 2-WNV NS1, indicating 30 site-specific amino acid mutations.

In Mosquito-borne ZIKV virus lineages linked to variable neurovirulence, NS1 structures exhibited lineage-specific changes at 9 AA. Comparison of NS1 monomers from Asian lineage (Brazil strain) and African lineage (Uganda strain) ZIKV isolates revealed AA changes in the central β-roll (1 AA), β-ladder (5 AA), and wing (3 AA) domain (Figure 3B, Supplementary Table S1). Of note, whilst the overall NS1 architecture is conserved, the Asian lineage is historically epidemiologically linked to congenital Zika syndrome,^41–43^ whereas African strains may cause fatal pathogenicity.^44^ Whilst the overall NS1 architecture is conserved, three AA changes in the surface-exposed Wing domain have not been investigated in the context of pathogenic outcomes. Together, this suggests that minor structural remodeling in the NS1 surface may underlie differences in tissue tropism and pathogenesis.

Structural variation in JEV NS1 from Genotype III (the highly pathogenic P20778 strain) and Genotype I (strain K94P05) exhibits conserved tertiary structures with 13 genotype-specific AA differences in the connector 2 loop (1 AA), the wing domain (4 AA), and the β-ladder (8 AA) (Figure 3C). These residues are positioned in proximity to putative glycosylation and oligomerization sites (Supplementary Table S1), potentially influencing NS1 dimerization or host immune interactions. Given that genotype I strains have displaced genotype III in several endemic regions, these may be related to transmissibility or immune escape.^45^

NS1 from WNV lineages show progressive changes within 29 AA, associated with high- and low-pathogenic strains. Structural comparison of NS1 from WNV Lineage 1a (highly pathogenic) and Lineage 2 (historically low-pathogenic, now emerging) revealed high conservation, with AA substitutions in each domain. The highest number was in the surface-exposed (wing) domain (11 AA). A total of 12 AA changes were in the β-ladder (Figure 3D). Notably, Lineage 2 NS1 exhibits substitutions in regions linked to lipid binding and ER membrane association, possibly linked with recent observations of increased neuroinvasive potential in clinical cases.^46^ These findings are consistent with the hypothesis that the evolution of NS1 within Lineage 2 may contribute to differences in its pathogenicity. Powassan virus is a tick-borne arbovirus endemic to North America.^47^ Comparison of Lineages 1 and 2 showed differences in NS1 sequences at only 1 AA in the β-roll (Phenylalanine [F] to Leucine [L] at position 247) (Supplementary Table S1).

These comparative structural analyses demonstrate that lineage- and strain-specific residues in NS1 cluster in functionally important domains across both tick- and mosquito-borne orthoflaviviruses. Substitutions at these regions may influence protein-cell membrane interactions, potentially contributing to differences in virulence and vector specificity, particularly in emerging strains with altered clinical outcomes. Moreover, these observations are based on sequence and structural analysis and provide a framework for future experimental investigation of the proposed functional effects.

### TBEV NS1 Interacts with Tight Junction Proteins in a Strain-Specific Manner

Given the established role of related arbovirus NS1 in disrupting endothelial integrity, we next investigated whether TBEV NS1 interacts with key TJPs, including JAM-A, JAM-B, JAM-C, claudin-5, and claudin-10. Structural predictions using AlphaFold3-multimer models were performed for both high- (Hypr) and low-pathogenic (Vs) NS1 variants, focusing on the extracellular domains (ectodomains) of the TJPs, which represent the biologically accessible interfaces for secreted or membrane-associated NS1. ZO-1, a cytoplasmic scaffold protein lacking an ectodomain, was included as a negative control. We also generated AF3 multimer binding models for previously reported interactions between ZIKA NS1 and claudins as controls for NS1 ^48^ (Table 1). We applied an ipTM threshold of ≥0.68 and a ranking score (calculated as 0.8 × ipTM + 0.2 × pTM) of ≥0.7 (Table 1), consistent with recent findings by Abulude et al. (2025) who demonstrated that ipTM scores in this range could be experimentally validated using the BACTH system and corresponded to biologically relevant complexes in *Bdellovibrio bacteriovorus*.^30^

**Table 1:** AlphaFold3 (AF3) Protein–Protein Docking Confidence Scores for Predicted Interactions Using ipTM and pTM Metrics

TBEV strain	TJ protein (human)	ipTM^a^	pTM^b^	Residual interacting	Ranking score^c^
Vs NS1	Claudin-5	0.71	0.8	7	0.728
Hypr NS1	Claudin-5	0.62	0.74		
Vs NS1	Claudin-10	0.49	0.64		
Hypr NS1	Claudin-10	0.7	0.79	12	0.718
Vs NS1	JAM-A	0.61	0.72		
Hypr NS1	JAM-A	0.68	0.78	10	0.7
Vs NS1	JAM-B	0.59	0.72		
Hypr NS1	JAM-B	0.63	0.74		
Vs NS1	JAM-C	0.54	0.67		
Hypr NS1	JAM-C	0.51	0.65		
Vs NS1	ZO-1	0.41	0.55		
Hypr NS1	ZO-1	0.38	0.52		
ZIKA 5K6K NS1	Claudin-5	0.76	0.83	1	0.774
ZIKA 5GS6 NS1	Claudin-5	0.76	0.84	3	0.776
ZIKA 5K6K NS1	Claudin-10	0.66	0.79	2	0.686
ZIKA 5GS6 NS1	Claudin-10	0.67	0.78	5	0.694

a ipTM (interface predicted TM score): Ranges from 0 to 1; higher values indicate better predictions.

b pTM (predicted TM score) ranges from 0 to 1; higher values are better.

c Ranking score: A weighted combination of ipTM and pTM (e.g., ranking_score = 0.8 × ipTM + 0.2 × pTM in AlphaFold’s methodology).

AF3 models predicted a confident interaction between JAM-A (Figure 4A) and Hypr NS1 (ipTM = 0.7; low interface PAE; Table 1 and Supplementary Table S2), involving the membrane-distal Ig-like domain of JAM-A engaging a hydrophobic groove on the NS1 dimer. Of interest, S142 and D236, predicted to mediate the Hypr NS1 interaction with JAM-A, are among the 22 amino acids that differ between Vs and Hypr strains.

**Figure 4:**
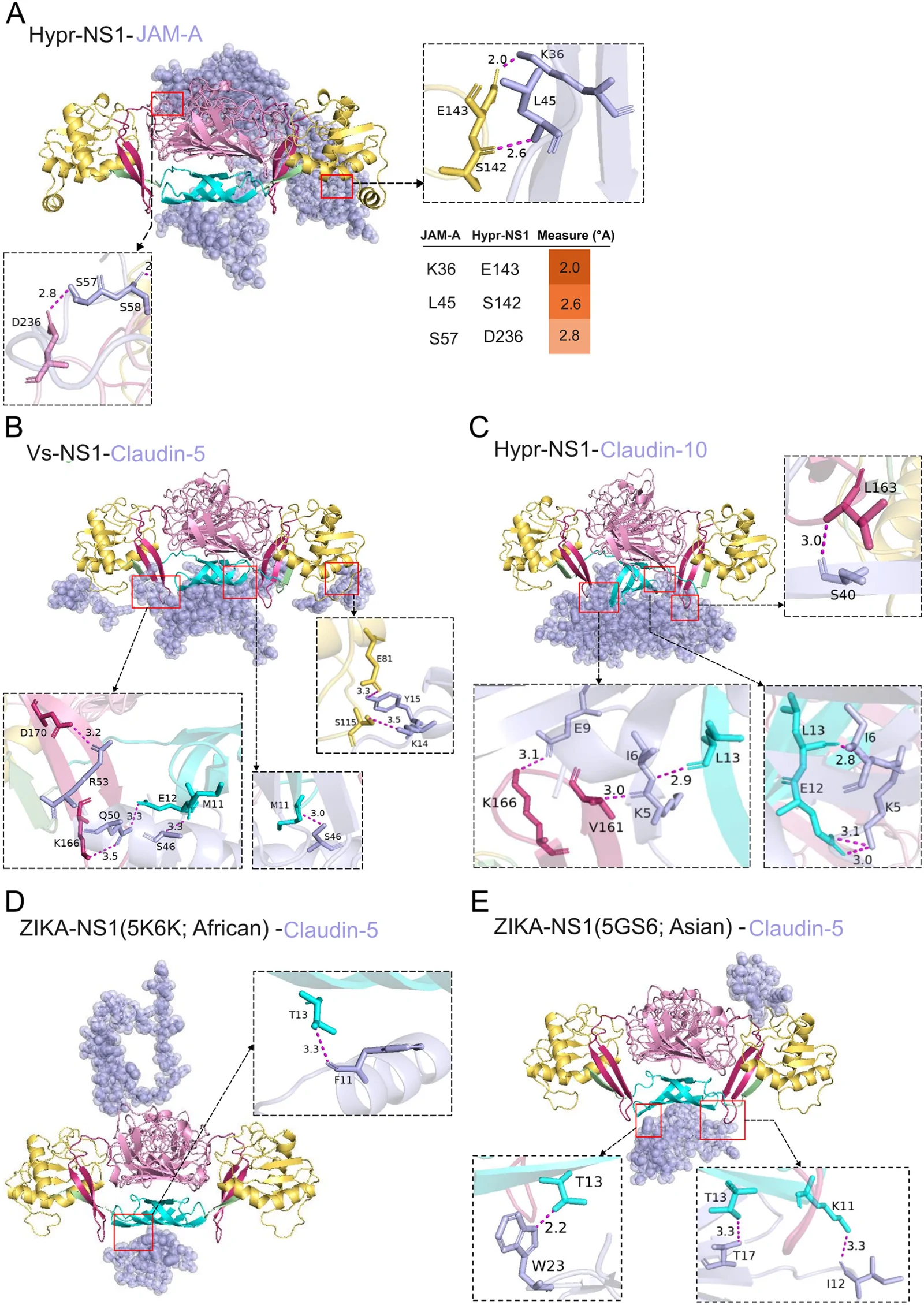
Predicted NS1–TJP interaction models generated using AlphaFold3 (AF3). (A) Interface-interacting residues are shown for TBEV Hypr-NS1 with JAM-A. The predicted selected distances (range: ≤2.0–2.8 Å) between amino acid interface residues of NS1 and human TJPs are shown. A heatmap indicates interaction distances; dark orange shorter distances; light orange longer distances. (B) Vs-NS1 interactions with Claudin-5 and (C) Hypr-NS1 with Claudin-10 are indicated. (D) The African (high-path) lineage of ZIKV and (E) Asian (low-path) NS1 interactions with Claudin-5 are shown. Predicted models with ipTM ≥ 0.68 and pTM ≤ 0.78 are included. NS1 domains are color coded as in Figure 1A.

Among other JAM family members, only JAM-B (Table 1) showed low-confidence interactions with Hypr NS1 (ipTM = 0.63), whilst JAM-C (ipTM ≥ 0.51) did not interact with either variant, indicating selective engagement rather than a general JAM-binding phenotype. As expected, no direct interactions were predicted between NS1 and ZO-1 (ipTM ≥ 0.38), consistent with ZO-1’s intracellular localization and supporting the specificity of the modeling approach.^49^

Claudin-5, which contains two short extracellular loops (ectodomains 1 and 2) similar to Claudin-10, did not form stable complexes with Hypr NS1 (Table 1 and Supplementary Table S3). Vs NS1 showed a modest interaction with Claudin-5 (ipTM = 0.71, Figure 4B). Only Hypr-NS1 (Figure 4C) was predicted to form interactions with Claudin-10 (ipTM = 0.7), suggesting potential Hypr/(Vs)-specific differences in claudin engagement. Parallel molecular docking of TBEV NS1 proteins (from Vs and Hypr strains) to Claudin-5 and Claudin-10 was performed using the HDOCK server, modeling both ectodomains 1 and 2 independently (Supplementary Table S4). Based on HDOCK’s reliability thresholds, only interactions with docking scores >200 were deemed as firm interactions. Similar to AF3 predictions, this shows possible interactions between Vs-NS1 and Claudin-5, as well as Hypr-NS1 and Claudin-10.

For proof-of-concept validation, we performed ELISA-based binding assays using recombinant NS1 and immobilized JAM-A or ZO-1. JAM-A was selected as a positive binder due to its greater intra- and extracellular accessibility, making it more amenable to antibody-based detection compared to claudins. Conformational analysis using far-UV circular dichroism (CD) spectroscopy (Figure 5A) confirmed the correct folding of TBEV NS1, with spectral characteristics comparable to those of commercially available NS1 (Figure 5B). Protein purity is shown in Figure 5C and D for Hypr and Vs, respectively. Hypr NS1 bound to JAM-A in a concentration-dependent manner at 0.5 μg/mL (Figure 5E), with an apparent K_D_ (K_D,app_) of 0.81 nM. Increasing the NS1 concentration to 1 μg/mL (Figure 5F) resulted in an K_D,app_ of 7.98 nM, whilst at 2 μg/mL (Figure 5G), the K_D,app_ was 310.38 nM. In contrast, Vs NS1 showed no binding across all tested concentrations (Figure 5H–J). Consistent with these findings, Hypr NS1 exhibited significantly higher OD_450_ values than Vs NS1 at both 0.25 μg/mL (Figure 5H) and 1 μg/Ml (Figure 5I). Binding assays with ZO-1 revealed no interaction for either NS1 protein (Figure 5J–L; 0.5, 1 or 2 μg/mL), consistent with the absence of predicted structural interfaces (Table 1). These data reinforce the lack of direct NS1-ZO-1 binding, despite the mislocalization reported in previous studies,^5,50^ and support the specificity of the assay while confirming the Hypr NS1–JAM-A interaction.

**Figure 5:**
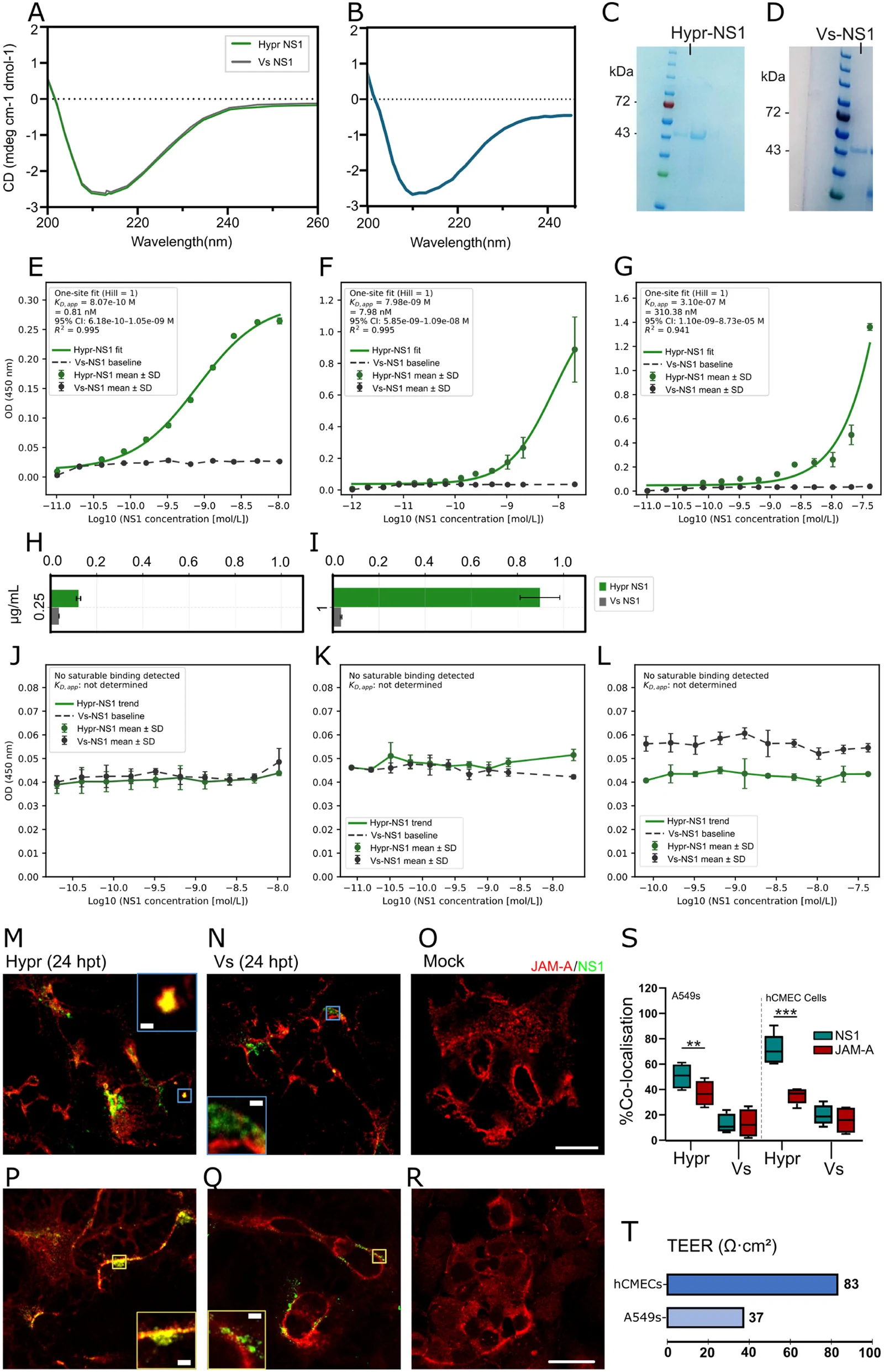
Strain-specific binding of TBEV NS1 to human TJPs. (A–D) Conformational analysis of refolded NS1 proteins. (A) Circular dichroism (CD) spectra of purified Hypr (green) and Vs (gray) NS1 proteins and (B) commercially sourced NS1 protein (The Native Antigen Company). SDS-PAGE analysis of purified (C) Hypr and (D) Vs NS1 protein elutions. Molecular weight standards are indicated. Proteins were resolved by SDS-PAGE under reducing conditions and stained with Coomassie Blue. (E–G) Binding curves of NS1 proteins from TBEV-Hypr (green) and TBEV-Vs (gray) to immobilized human JAM-A, measured by ELISA across three independent experiments. NS1 at starting concentrations of (E) 0.5 μg/mL, (F) 1 μg/mL, or (G) 2 μg/mL was titrated in 2-fold serial dilutions. Data points represent mean OD450 ± SD from duplicate wells. Apparent equilibrium dissociation constants (K_D,app_) were estimated by nonlinear least-squares regression of untransformed molar concentrations using a one-site specific binding model. Fitted curves and K_D,app_ values are shown. Quantification of NS1 binding to JAM-A at (H) 0.25 μg/mL and (I) 1 μg/mL. Bars represent mean OD_450_ ± SD. (J–L) Binding curves of Hypr and Vs NS1 proteins to immobilized human ZO-1, measured by ELISA across three independent experiments. NS1 was used at starting concentrations of (J) 0.5 μg/mL, (K) 1 μg/mL, or (L) 2 μg/mL and titrated in 2-fold serial dilutions. Data points represent mean OD450 ± SD from duplicate wells. Concentration–response data showed no evidence of saturable binding suitable for equilibrium binding analysis; consequently, K_D,app_ values were not determined. Trend lines are shown for visualization only. (M–R) Immunofluorescence analysis of TBEV NS1 and JAM-A in transfected A549 cells ([M], Hypr; [N], Vs; [O], mock) and hCMEC/D3 cells ([P], Hypr; [Q], Vs; [R], mock) at 24 h post-transfection. Cells were fixed with 4% paraformaldehyde and stained with anti-NS1 (R&D Systems; green) and anti-JAM-A (Bio-Techne; red) antibodies before imaging using a Zeiss LSM780 or Olympus FV3000 confocal microscope. Scale bars: 20 μm; inset images, 1 μm. (S) Subcellular colocalization analysis of TBEV NS1 and JAM-A in A549 and hCMEC/D3 cells. Box plots show the percentage of NS1 colocalized with JAM-A (green) or JAM-A colocalized with NS1 (red). Colocalization was quantified in 230 transfected cells from two independent experiments using Manders’ overlap coefficients (M1 and M2) calculated in Fiji with the JACoP plugin. M1 represents the fraction of NS1 signal overlapping with JAM-A, whereas M2 represents the fraction of JAM-A signal overlapping with NS1. Coefficient values range from 0 to 1. Statistical significance was assessed using one-way ANOVA followed by Bonferroni’s multiple-comparisons test. *p* < 0.01; ****p < 0.0001. (T) Trans-epithelial/endothelial electrical resistance (TEER) measurements of A549 and hCMEC/D3 monolayers obtained 25 h after seeding using an EVOM2 epithelial voltohmmeter fitted with STX electrodes. TEER values are expressed as Ω·cm^2^.

To further investigate the observed Hypr/(Vs) NS1–JAM-A interactions, IF analyses was performed in A549 cells or hCMEC/D3 cells, a human cerebral endothelial cell line that is permissive to TBEV infection,^7^ transfected with Hypr or Vs replicons (Figure 5M–N or P–Q) for 24 h, or mock (Figure 5O–R), as described previously.^36^ JAM-A is shown in red and NS1 in green. Colocalization is indicated by yellow fluorescence in the merged images. Hypr NS1 exhibited substantial colocalization with JAM-A in both A549 (Figure 5M) and hCMEC/D3 cells (Figure 5P), whilst minimal colocalization was observed in Vs replicon-transfected cells (Figure 5N,Q). Mock-transfected controls showed no NS1 signal (Figure 5O,R). Quantification analyses (Figure 5S) revealed significantly higher NS1–JAM-A colocalization in Hypr-transfected cells compared to Vs-transfected cells, with 50–60% and 70–80% colocalization observed in A549 and hCMEC/D3 cells, respectively. hCMEC/D3 cells exhibited a significantly (*p* = 0.0008) greater degree of NS1–JAM-A colocalization than A549 cells. To assess monolayer integrity, TEER was measured 24 h postseeding (Figure 5T). hCMEC/D3 monolayers exhibited a value of 83 Ω·cm^2^, whilst A549 monolayers exhibited a value of 37 Ω·cm^2^, indicating greater barrier integrity in hCMEC/D3 cells and further supporting the potential relevance of this interaction in endothelial barriers.

Of note, due to technical limitations, glycosylation-associated effects of NS1-TJP binding could not be predicted using our *in silico* models. However, ELISA assays showed no significant differences in NS1–TJP binding in NS1 expressed in *E. coli* *or* mammalian HEK293 human cells (Native Antigen). Additionally, the binding affinity reported represents an apparent K_D_ derived from an ELISA-based assay and may be affected by assay-specific factors, including protein immobilization, avidity effects, and signal amplification. Nevertheless, ELISA data were corroborated by our immunofluorescence analyses, which demonstrated a consistent binding pattern.

Together, these data indicate that TBEV NS1 engages JAM-A in a strain-specific manner, with only the virulent Hypr NS1 variant showing direct binding and colocalization in hCMEC/3D cells. These findings highlight a potential role for the NS1–JAM-A interaction in the disruption of the BBB during infection with pathogenic strains of TBEV (graphical illustration).

## Discussion

Despite the high conservation of orthoflavivirus NS1 across both tick-borne and mosquito-borne members, emerging evidence suggests divergent functional roles during pathogenesis shaped by vector lineage, host tropism, and strain-specific viral factors.^5,6,14,27,28,50–52^ This study provides mechanistic insight into how NS1 interactions could relate to strain-specific disruption of the endothelial barrier in TBEV, focusing on NS1 interactions with BBB TJs. A key finding is the direct binding observed between NS1 from the neurovirulent Hypr strain and the adhesion molecule JAM-A, which was not the case for the low-virulence Vs strain. This differential binding was validated using biochemical (ELISA) and immunofluorescence assays (Figure 5) in physiologically relevant brain microvascular endothelial hCMEC/3D cells that represent a model of the human BBB.^7^

Given that JAM-A has an established role in maintaining BBB integrity and regulating immune cell transmigration,^53,54^ its engagement by Hypr NS1 may contribute to paracellular permeability and endothelial destabilization. This points to a targeted mechanism of BBB disruption, distinct from the generalized vascular leakage observed in mosquito-borne DENV and ZIKV, in which NS1 induces endothelial dysfunction through pattern-recognition receptor activation (e.g., TLR4) or complement pathways.^6,12^

Unlike DENV NS1, which interacts with ZO-1 and claudins to broadly destabilize endothelial junctions,^55^ TBEV NS1 appeared to selectively engage claudins as indicated by the *in silico* AF3 model (Figure 4). This suggests that BBB disruption in tick-borne orthoflaviviruses may rely on precise modulation of the endothelial interfaces critical for CNS entry, as opposed to pan-junctional disassembly. Our data also raise the intriguing hypothesis that the Hypr NS1–JAM-A interaction may be involved in neuroinvasion. Whether this represents a causal gateway or a consequence of systemic vascular damage, however, remains to be determined.

Structurally, NS1 is a conserved dimer/trimer comprising β-roll, wing, and β-ladder domains.^10,11^ Comparative analyses revealed that the 22 AA differences between Hypr and Vs NS1 localize to the wing and β-ladder domains implicated in host membrane interactions and immune modulation. For other orthoflaviviruses, these domains mediate lipid binding, immune complex formation, and cell surface anchorage.^56,57^ The clustering of strain-specific substitutions in these surface-exposed regions may support a model in which minor sequence variations may reshape host interaction profiles, in the absence of altering the architecture of NS1.

This concept aligns with the broader patterns observed during orthoflavivirus evolution. For example, minor substitutions in NS1 from ZIKV (Asian vs African lineages) or WNV (lineage 1 vs 2) are sufficient to alter neurotropism, likely via changes in host protein or membrane interactions.^46,58^ Our findings suggest similar adaptive pressures for tick-borne orthoflaviviruses, where subtle NS1 modifications may fine-tune endothelial engagement and influence the neuroinvasive potential.

The absence of JAM-A binding by Vs-NS1 underscores the existence of alternative CNS entry mechanisms. Although Vs is less neurovirulent in murine models, human infections can result in persistent neurological symptoms and chronic inflammation.^25,26^ This suggests that Vs pathogenesis may involve more gradual neuroinvasiveness via mechanisms that do not require direct NS1-JAM-A interactions (e.g., immune cell trafficking or inflammation-induced paracellular leak).

Interestingly, our AF3 model predicted that Vs NS1 exhibits modest binding to Claudin-5 and Hypr NS1 to Claudin-10, key components of BBB tight junctions responsible for regulating paracellular ion flux and maintaining endothelial barrier integrity.^53,59^ The physiological relevance of these interactions and the potential involvement of post-translational modifications such as glycosylation in their regulation, remains unknown. Claudin extracellular loops are short and structurally constrained. Direct NS1–claudin interactions have not been demonstrated *in vivo*. It is more plausible that claudin disassembly during TBEV infection results from cytokine-driven inflammatory signaling, as observed in DENV where NS1 triggers TLR4-mediated endothelial activation and downstream production of TNF-α and IL-6, leading to tight junction destabilization.^60^ ZIKA NS1 has been shown to suppress Claudin-5 expression in human brain microvascular endothelial cells, contributing to BBB breakdown.^52^ Although that study did not assess direct NS1–Claudin-5 binding, our AF3 multimer modeling predicted moderate binding of both African- and Asian-lineage NS1 proteins to Claudin-5 (Figure 4D–E and Table 1 and Supplementary Table S5; ipTM = 0.76), consistent with strains previously associated with BBB disruption.^3,41,61^

Furthermore, AF3 predicted distinct claudin-binding profiles for Hypr NS1 and Vs NS1, and their interaction networks also differed substantially. The Hypr NS1–Claudin-10 complex exhibited 12 predicted contacts, compared with 7 for the Vs NS1–Claudin-5 complex (Supplementary Table S3). Similar strain-dependent differences were observed in ZIKV NS1 models (Supplementary Table S5), despite large conservation of their interface residues. These observations suggest that variation in the extent and stability of protein–protein interactions may contribute to strain-specific binding properties, although experimental validation is required to substantiate this conclusion.

In line with this study, a recent study demonstrated NS1-TNF-α-mediated increased endothelial permeability in TBEV-infected human lung microvascular endothelial cells. Direct NS1–TJP interactions were not investigated, and only the Far Eastern subtype Sofjin strain was studied,^62^ limiting mechanistic comparison. The findings were, however, consistent with our data and suggest that NS1-mediated pathogenicity may be regulated by multiple strain-specific mechanisms, enabling differential clinical outcomes. Similar phenomena have been reported for other neurotropic viruses, such as JEV, where cytokine-mediated modulation of claudins as opposed to direct virus protein binding, appears to drive changes in BBB permeability.^63^

In summary, we present evidence from predictive modeling and *in vitro* binding assays suggesting that TBEV NS1 may engage endothelial junction proteins, particularly JAM-A, in a strain-specific manner. Through the integration of computational structural predictions, evolutionary sequence analysis, and *in vitro* validation, we offer a framework to compare viruses with differential pathogenesis. While claudins also represent key TJPs identified as potential NS1 binders *in silico*, Claudin-5 and Claudin-10 are predominantly localized within intercellular junction complexes, which can limit their accessibility *in vitro*. Future studies will investigate their role in conjunction with the *in vivo* mechanisms of NS1-mediated junction disruption to assess how naturally occurring mutations influence these interactions in emerging strains. Given the expanding geographical range of both tick- and mosquito-borne orthoflaviviruses driven by climate change,^64–66^ monitoring NS1 sequence evolution may provide early indicators of shifts in virulence or transmission potential, informing diagnostic development and vaccine design.

## Supplementary Material



## Notes

Supplementary Table S1: Comparison of NS1 amino acid changes between high- and low-pathogenic orthoflavivirus strains (PDF); Supplementary Table S2: Residue interactions between Hypr NS1 and JAM-A (PDF); Supplementary Table S3: Residue interactions between Vs NS1-Claudin-5 and Hypr-NS1 and Claudin-10 (PDF); Supplementary Table S4: Comparison of HDOCK-predicted TBEV NS1 binding to human TJPs; Supplementary Table S5: Residue interactions between ZIKV NS1 and Claudin-5 (PDF)
