# Supplementary material for: Interactions between Tick-Borne Encephalitis Virus Nonstructural Protein 1 and Blood–Brain Barrier Tight Junction Proteins: Potential Clues to Strain-Specific Neuropathogenicity
Source: ACS Omega. 2026 Sep 22;11(39):58584–98. doi: 10.1021/acsomega.6c00564 (PMC13647419; doi:10.1021/acsomega.6c00564)
Supplement: Supplementary file 1 [file ao6c00564_si_001.pdf]

Interactions between tick-borne encephalitis virus non-structural protein 1 and blood-brain barrier tight junction proteins: potential clues to strain-specific neuropathogenicity

*Jarinya Chaopreecha[#], Jordan Liburd[α], Shahid Khalid[#], Songeziwe Ntsimango[ɵ], Ian M Jones[∞, *], Jennifer H. Tomlinson[α, *], Niluka Goonawardane [1,≠, ±]*

Affiliation:

[#] School of Molecular and Cellular Biology Faculty of Biological Sciences, University of Leeds, Leeds, LS2 9JT, UK.

[ɵ] Molecular Sciences Institute, School of Chemistry, University of the Witwatersrand, PO Wits 2050, Johannesburg, South Africa.

[∞] School of Biological Sciences, University of Reding, Reading, UK.

[α] Astbury Centre for Structural Molecular Biology, School of Molecular and Cellular Biology Faculty of Biological Sciences, University of Leeds, Leeds, LS2 9JT, UK.

[≠] Department of Biology, University of Oxford, Life and Mind Building, South Parks Road, Oxford, OX1 3EL, UK.

* Equally contributed to this study.

[±] Corresponding author: Dr Niluka Goonawardane. niluka.goonawardane@biology.ox.ac.uk

* Equally contributed to this study.

---

[1] Astbury Centre for Structural Molecular Biology, School of Molecular and Cellular Biology Faculty of Biological Sciences, University of Leeds, Leeds, LS2 9JT, UK.

Supplementary Table S1: Comparison of amino acid changes in the NS1 protein of tick-borne encephalitis virus (TBEV), Zika virus (ZIKV), Japanese encephalitis virus (JEV), West Nile virus (WNV), and Powassan virus (POWV) between prototype high- and low-pathogenic strains.

NS1 Domain	Position	TBEV	
		**Hypr (High pathogenic)**	**Vs (Low pathogenic)**
**Wing**	54	S	N
	57	V	I
	71	S	A
	83	E	D
	89	M	V
	93	F	L
	121	H	Q
	141	Q	G
	142	S	N
**β - ladder**	194	I	V
	203	R	K
	205	M	V
	236	D	E
	262	S	A
	271	H	Y
	272	T	S
	277	I	T
	285	T	K
	289	N	S
	291	K	D
	316	A	T
	319	M	L

NS1 Domain	Position	POWV	
		**Lineage 1 (High pathogenic)**	**Lineage 2 (Low pathogenic)**
**β - ladder**	247	F	L

NS1 Domain	Position	ZIKV	
		**Asin strain (High pathogenic)**	**Uganda strain (Low pathogenic)**
**β - roll**	21	V	I
**Wing**	52	D	E
	69	R	K
	146	K	E
**β - ladder**	191	K	R
	194	V	A
	236	I	V
	264	M	V
	286	H	Y

NS1 Domain	Position	JEV	
		**Vellore (High pathogenic)**	**K94P05 (Low pathogenic)**
**Wing**	50	K	Q
	69	A	S
	131	T	S
	146	H	R

**Connector 2**	174	S	N
**β - ladder**	205	Y	L
	219	I	V
	250	K	R
	272	I	L
	274	L	P
	275	D	G
	276	F	L
	297	V	I

**NS1 Domain**	**Position**	**WNV**	
		**High path (Lineage 1a)**	**Low path (Lineage 2)**
**β - roll**	8	S	G
**Connector 1**	33	Y	F
**Wing**	43	K	R
	50	K	A
	70	V	I
	93	E	N
	98	S	A
	104	T	A
	112	I	M
	122	L	I
	134	V	I
	140	K	E
	145	Q	A
**Connector 2**	152	L	M
	169	K	R
	170	V	I
	173	S	T
**β - ladder**	191	L	M
	193	I	V
	204	R	G
	235	I	V
	244	V	I
	285	L	I
	287	E	D
	296	T	A
	322	Y	F
	325	D	E
	326	S	N
	337	Q	T

Supplementary Table S2: Residue interactions between Hypr NS1 and JAM-A.

Residue		Distance (Å) ±	Bond †
Hypr NS1	JAM-A		
E143	K36	2	SB (ionic interaction)
K47	N90	2.3	HB
S142	L45	2.6	Weak HB / polar–hydrophobic contact
S142	T43	3.1	HB
D233	S3	2.6	HB
F50	R32	2.8	Cation–π interaction
R148	E34	2.8	SB (ionic interaction)
A352	S3	3	vdW
R257	G5	3.1	HB
D236	S57	2.8	HB

± *Residue pairs were ranked by shortest interatomic distance. All distances are reported in Ångströms (Å).*

† *Abbreviations used in this table:* van der Waals = vdW; *SB = Salt bridge; HB = Hydrogen bond.*

Supplementary Table S3: Residue interactions between Vs NS1- Claudin-5 and Hypr-NS1 and Claudin-10.

Residue		Distance (Å) ±	Bond †	Residue		Distance (Å) ±	Bond †
**Vs NS1**	**Claudin-5**			**Hypr NS1**	**Claudin-10**		
M11	S46	3	Weak polar contact	K166	E9	2.7	SB
D170	R53	3.2	SB	L13	I6	2.8	vdW
E81	Y15	3.3	HB	L13	I6	2.9	vdW
M11	S46	3.3	HB	V161	K5	3	vdW
E12	Q50	3.3	HB	E12	K5	3	SB
S115	K14	3.5	HB	E12	T7	3	HB
K166	Q50	3.5	HB	E158	K5	3	SB
				E158	T7	3	HB
				L163	S40	3	weak polar contact
				E12	T7	3.1	HB
				K166	E9	3.1	SB
				L163	A3	3.1	vdW

± *Residue pairs were ranked by shortest interatomic distance. All distances are reported in Ångströms (Å).*

† *Abbreviations used in this table:* van der Waals = vdW; *SB = Salt bridge; HB = Hydrogen bond.*

Supplementary Table S4: Strain-specific binding affinities for TBEV NS1 interactions with full-length or isolated ectodomains for selected human TJPs determined by HDOCK server.

**NS1-TJP Complex**	**Docking Score***	**ΔG (25 °C) [kcal/mol]**	**$K_{D,app}$ (25 °C) [M]**	**ΔG (40 °C) [kcal/mol]**	**Kd (40 °C) [M]**	**Fold Change (25 °C / 40 °C)**
Vs NS1-Claudin-5 full-length	-319.37	-319.37	7.95E-235	-319.37	1.30E-223	6.12E-12
Hypr NS1-Claudin-5 ectodomain 1	-265.4	-265.4	2.89E-195	-265.4	6.01E-186	4.80E-10
Vs NS1-Claudin-5 ectodomain 2	-165.77	-165.77	3.09E-122	-165.77	2.04E-116	1.51E-06
Hypr NS1-Claudin-10 full-length	-287.44	-287.44	2.02E-211	-287.44	2.50E-201	8.08E-11
Hypr NS1-Claudin-10 ectodomain 1	-267.01	-267.01	1.91E-196	-267.01	4.52E-187	4.22E-10
Hypr NS1-Claudin-10 ectodomain 2	-138.96	-138.96	1.39E-102	-138.96	1.05E-97	1.32E-05

**Docking Score: The docking scores are calculated by the knowledge-based iterative scoring function ITScorePP or ITScorePR. A more negative docking score means a more possible binding model, (≥ 200= higher binding).*

Supplementary Table S5: Residue interactions between ZIKV NS1 and Claudin-5.

Residue		Distance (Å) ±	Bond †	Residue		Distance (Å) ±	Bond †
**ZIKA 5K6K NS1**	**Claudin-5**			**ZIKA 5GS6 NS1**	**Claudin-5**		
T13	F11	3.3	vdW	K11	I12	2.2	Weak vdW
				T13	T17	3.3	HB
				T13	W23	3.3	HB

± *Residue pairs were ranked by shortest interatomic distance. All distances are reported in Ångströms (Å).*

† *Abbreviations used in this table:* van der Waals = vdW; *SB = Salt bridge; HB = Hydrogen bond.*
